# Defining a Novel *RPGR* Phenotype of Sector Retinitis Pigmentosa With Cone Dystrophy

**DOI:** 10.1167/iovs.67.10.24

**Published:** 2026-08-10

**Authors:** Maram E. A. Abdalla Elsayed, Omar A. Mahroo, Andrew Webster, Stephen H. Tsang, João Pedro Marques, Elias Traboulsi, Tien-En Tan, Camiel J. F. Boon, Cristina Martinez-Fernandez de la Camara, Beau J. Fenner, Vincenzo Barone, Hélène Dollfus, Robert E. MacLaren

**Affiliations:** 1 Oxford Eye Hospital, Oxford University Hospitals NHS Foundation Trust, Oxford, United Kingdom; 2 Nuffield Laboratory of Ophthalmology, Department of Clinical Neurosciences, University of Oxford, Oxford, United Kingdom; 3 NIHR Biomedical Research Centre at Moorfields Eye Hospital and the UCL Institute of Ophthalmology, London, United Kingdom; 4 Edward S. Harkness Eye Institute, Department of Ophthalmology, Columbia University Irving Medical Center/New York-Presbyterian Hospital, New York, New York, United States; 5 Ophthalmology Department, Hospitais da Universidade de Coimbra, Unidade Local de Saúde de Coimbra, Coimbra, Portugal; 6 Cole Eye Institute, Cleveland Clinic, Cleveland, Ohio, United States; 7 Singapore Eye Research Institute, Singapore National Eye Centre, Singapore, Singapore; 8 Department of Ophthalmology, Amsterdam University Medical Centre, Amsterdam, the Netherlands; 9 Ophthalmology Complex Operative Unit, Fondazione Policlinico Universitario Campus Bio-Medico, Rome, Italy; 10 Scheie Eye Institute, Department of Ophthalmology, University of Pennsylvania Perelman School of Medicine, Philadelphia, Pennsylvania, United States; 11 ERN-EYE Centre de Référence Pour les Affections Rares en Génétique Ophtalmologique, Institut de Génétique Médicale d'Alsace, FSMR SENSGENE, Hôpitaux Universitaires de Strasbourg, Strasbourg, France

**Keywords:** *RPGR*, cone dystrophy, inherited retinal degeneration, gene therapy, genetics

## Abstract

**Purpose:**

*RPGR^ORF15^*-associated retinal degeneration is characterized by clinical and genetic heterogeneity: Proximal mutations typically result in rod–cone dystrophy, distal mutations in cone-dominated disease, and mutations within open-reading frame 15 (ORF15) in either phenotype. This study characterizes an intermediate phenotype in which patients exhibit a combination of cone dystrophy and incomplete (sectoral) rod–cone dystrophy associated with mutations in the ORF15 region and explores potential mechanistic explanations.

**Methods:**

A multinational, multicenter, observational, cross-sectional case series was conducted using databases from *RPGR*-related retinal dystrophy clinical trial referral centers. Patients with molecularly confirmed *RPGR*-related cone dystrophy or *RPGR*-related cone–rod dystrophy were studied. Individuals exhibiting a mixed phenotype of cone dystrophy and sectoral retinitis pigmentosa were identified. In silico analyses assessed the impact of identified mutations on RPGR transcript expression and protein structure.

**Results:**

Fourteen patients exhibited a cone dystrophy phenotype with bilateral, symmetrical regions of outer retinal atrophy distributed along the inferior vascular arcades and extending nasally. All harbored ORF15 mutations within a defined transitional zone. All of the mutations were predicted to produce truncated proteins with partial or complete loss of function. Additionally, several were predicted to disrupt splicing regulatory elements.

**Conclusions:**

An intermediate phenotype consisting of a cone dystrophy with sectoral retinitis pigmentosa development was characterized. These patients may benefit from full-length *RPGR* gene therapy. Furthermore, we demonstrate that this rare presentation closely resembles the phenotype observed in some patients with loss-of-function *TTLL5*-associated cone dystrophy with sectoral involvement.

With retinitis pigmentosa GTPase regulator (*RPGR*) gene–related rod–cone dystrophies being the focus of two current phase III clinical trials (NCT04671433 and NCT04850118), deep phenotyping is imperative for improving diagnostic accuracy, considering patient eligibility, and understanding disease mechanisms of this currently untreatable disease. Pathogenic variants in the *RPGR* gene represent a significant cause of visual impairment, accounting for nearly 5% of all instances of retinal degeneration.^1^ More than two-thirds of patients with *RPGR*-related retinopathy have a severe, early-onset form of X-linked rod–cone dystrophy; however, *RPGR*-related retinopathy exhibits significant phenotypic heterogeneity, with some mutations resulting in a cone or cone–rod phenotype.^2^

The *RPGR* gene, located on chromosome Xp11.4, undergoes extensive alternative splicing and the two major isoforms are RPGR*^ex1^^–^^19^* (815 amino acids) and *RPGR*^ORF15^ (1152 amino acids). Of these, RPGR^ORF15^ is the retina-specific isoform and is most frequently associated with inherited retinal degenerations (IRDs). RPGR^ORF15^ shares the first 15 exons with RPGR*^ex1−19^*, then parts of intron 15 are included into the transcript to generate the exon open-reading frame 15 (ORF15).^3^ This isoform is comprised of the shared N-terminal regulator of chromosome condensation 1 (RCC1)-like domain found in the constitutive *RPGR^ex1−19^* isoform, followed by a unique C-terminal region encoded by exon ORF15 (Fig. 1).^3^ The ORF15 region encodes a glutamate/glycine–rich domain, which is followed by the nonrepetitive and highly conserved basic domain, rich in basic amino acids at the extreme C terminus. The RPGR^ORF15^ isoform is localized to the base of the connecting cilium of rod and cone photoreceptors, a structurally and functionally specialized region that bridges the inner and outer segments and facilitates microtubule-based intraflagellar transport. Within this compartment, RPGR^ORF15^ interacts with a range of cilia-associated and trafficking proteins, including RPGR interacting protein 1 (RPGRIP1), phosphodiesterase 6D (PDE6D), centrosomal protein 290 (CEP290), and tubulin tyrosine ligase-like 5 (TTLL5), all of which are integral to the regulation of photoreceptor protein transport and ciliary maintenance.^4^^–^^9^ The C-terminal region of RPGR^ORF15^ binds directly to TTLL5, a member of the tubulin tyrosine ligase-like (TTLL) enzyme family, which mediates glutamylation—a post-translational modification involving the addition of glutamate side chains to 11 glutamate-rich consensus motifs within RPGR^ORF15^.^10^ This modification is postulated to influence the structural conformation, stability, and protein–protein interactions of RPGR^ORF15^, in a manner analogous to the modulation of microtubule dynamics through tubulin glutamylation.^11^

**Figure 1.**
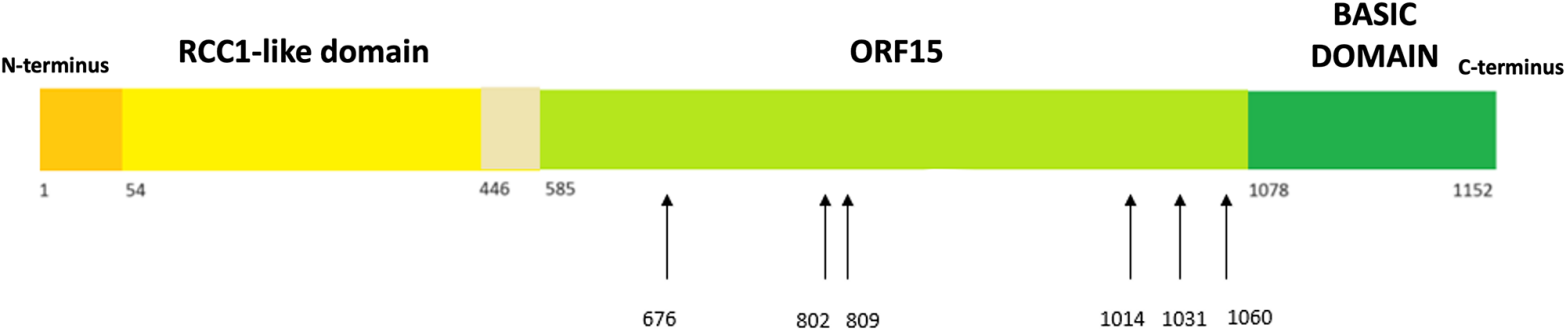
Schematic of the RPGR^ORF15^ protein illustrating the major domains. The *black arrows* indicate pathogenic RPGR variants associated with the cone dystrophy and sectoral rod–cone phenotype. These variants are distributed along RPGR^ORF15^: 676, c.2027_2039del, p.(Lys676Thrfs17); 802, c.2405_2406delAG, p.(Glu802Glyfs32); 809, c.2426_2427delAG, p.(Glu809Glyfs25); 1014, c.3040delG, p.(Glu1014Argfs75); 1031, c.3092delA and c.3092_3093delAG, p.(Glu1031Glyfs58/47); and 1060, c.3178_3179delGA, p.(Glu1060Argfs18).

Although disease-causing mutations have been identified throughout the *RPGR^ORF15^* gene, most are concentrated within the ORF15 region, a known mutational hotspot responsible for up to 80% of *RPGR*-related retinal dystrophies. This stretch of DNA, spanning nucleotides 2184 to 3234, is unusually rich in purines, with nearly 98% of the sequence composed of adenine (A) and guanine (G).^12^ Such a composition can promote unstable DNA structures that compromise the accuracy of DNA replication and transcription, increasing the likelihood of harmful mutations. The most common pathogenic *RPGR^ORF15^* variants are frameshift mutations that result mainly from the deletion of one or more nucleotides. The majority of frameshift variants within ORF15 are predicted to evade nonsense-mediated decay (NMD), resulting in the production of stable but truncated proteins that may have either loss- or gain-of-function consequence.^12^ Deletions in this purine-rich region not only shift the reading frame but can also disrupt normal splicing. They may activate cryptic splice sites or alter the balance between exonic splicing enhancers (ESEs) and exonic splicing silencers (ESSs), leading to aberrant transcript processing. Because ORF15 spans exon 15 and part of intron 15, preserving the constitutive splice donor site, such disruptions can result in partial exon skipping or abnormal inclusion of intronic sequences, ultimately producing incompletely truncated or misfolded proteins.

Previous studies have shown that distal mutations in *RPGR^ORF15^* result in a cone-dominant phenotype, whereas upstream mutations in the majority of the gene result in a rod-dominant phenotype.^13^^–^^16^ Evidence suggests that a functional N-terminal region containing the RCC1-like domain is likely to have a more important role in rods, whereas an intact distal part of the protein and its glutamylation is likely to serve a more dominant function in cones.^11^ The watershed zone described in those previous studies is comprised of most of the ORF15 region, where mutations can lead to either rod- or cone-dominated phenotypes. We hypothesized that patients with mutations in this zone may have a sectoral rod–cone dystrophy along with a cone dystrophy. In this study, we observed a previously unreported retinal phenotype characterized by the coexistence of clinical features typical of a cone dystrophy and of sectoral retinitis pigmentosa. This phenotype exhibits a distinctive structural and functional profile that may represent a transitional state between classic cone–rod dystrophy and cone-only dystrophy. These findings are of particular clinical relevance, as individuals with this phenotype may be well suited for inclusion in ongoing phase III gene therapy trials. Furthermore, we demonstrated that this rare presentation closely resembles the phenotype observed in some patients with loss-of-function *TTLL5*-associated cone dystrophy with sectoral involvement.

## Methods

This was a cross-sectional, observational case series conducted at international centers specializing in IRDs. Eligible patients were patients confirmed to harbor pathogenic variants in *RPGR*, with a clinical diagnosis of cone dystrophy or cone–rod dystrophy. The intermediate RPGR-associated phenotype was defined by central macular involvement associated with sectoral peripheral retinal degeneration, without progression to generalized rod–cone dystrophy on available longitudinal follow-up. Patients with full rod–cone or cone-only phenotypes without sectoral features were excluded. Patients with other plausible retinal diseases and disease-associated variants were excluded. Genetic testing results, fundus imaging, optical coherence tomography (OCT), ophthalmological examination findings, and full-field electroretinography, when available, were obtained. Fundus imaging consisted of color fundus imaging (Topcon Medical Systems, Oakland, NJ, USA) or pseudocolor imaging (Optos, Dunfermline, Scotland). OCT imaging was obtained using either SPECTRALIS (Heidelberg Engineering, Heidelberg, Germany) or CIRRUS (Carl Zeiss Meditec, Dublin, CA, USA) devices. Where available, visual fields were collected. All imaging was reviewed by at least three IRD specialists, who were not masked to genotype. *RPGR* mutations were identified by targeted next-generation sequencing techniques, and putative pathogenic variants were confirmed by Sanger sequencing. Full-field electroretinography (ERG) was performed according to the International Society for Clinical Electrophysiology of Vision (ISCEV) Standard methodology. Additionally, because TTLL5 interacts with the C-terminus of RPGR to facilitate post-translational modification, we reviewed patients with *TTLL5* mutations to compare that phenotype to the RPGR intermediate phenotype we describe. Data were retrospectively collected from institutional databases. The study was approved by the appropriate institutional review boards at each participating center, and all procedures were conducted in accordance with the tenets of the Declaration of Helsinki.

The impact of the mutations analyzed in this study on RNA processing was evaluated using a range of bioinformatic tools. As all mutations introduce a premature stop codon, the sequences of the resulting truncated protein variants were aligned using Geneious Prime 2024.0.4. A color-coded map was generated to visualize the altered amino acid composition. NMDEsc predictor was used to confirm whether the mutant transcripts are likely to escape nonsense-mediated mRNA decay and remain stable for translation. The Human Splicing Finder (HSF) Pro tool was used to assess the impact of each mutation on splicing elements, including splice sites, branch points, and auxiliary regulatory sequences such as exonic and intronic splicing enhancers or silencers.

## Results

Fourteen patients with *RPGR* variants, including 13 hemizygous males and one heterozygous female, with features combining a cone and a sectoral rod–cone dystrophy phenotype, were identified. At the most recent examination, 11 of 14 patients (79%) were older than 40 years, with a median age of 52 years and an age range of 15 to 91 years.

**Figure 2.**
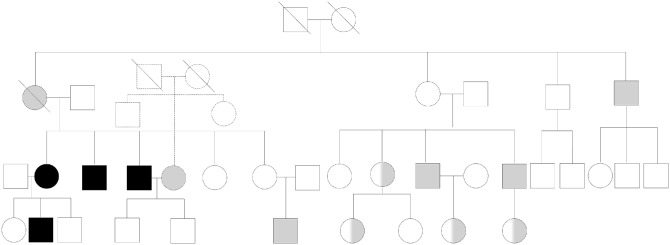
Pedigree of the family carrying the RPGR c.3092delA, p.(Glu1031Glyfs*58) variant. The pedigree demonstrates X-linked transmission of the familial *RPGR* variant, with multiple affected individuals across generations. *Black-filled symbols* indicate patients with the intermediate RPGR-associated phenotype described in this study (patients 7, 9, 10, and 11), including one heterozygous female. *Gray-filled symbols* indicate affected relatives with a conventional *RPGR*-associated retinal phenotype. *Half-gray* *symbols* indicate genetically confirmed heterozygous female carriers. *Open symbols* indicate unaffected or untested individuals.

All seven reported variants in this cohort consisted of one or two base-pair deletions, leading to frameshift mutations in the ORF15 region. The most common variant in this cohort was RPGR c.3092delA, p.(Glu1031Glyfs*58) in six of 14 pedigrees (43%), followed by RPGR c.2405_2406delAG, p.(Glu802Glyfs*32) in three pedigrees (21%). The remainder of the variants were each observed in a single patient (Table). Patients 7, 9, 10, and 11 belonged to the same pedigree, whereas patients 8 and 12 carried the same variant but were from unrelated families. (Fig. 2).

**Table.** Summary of the Patient Characteristics and Predicted Impacts of RPGR^ORF15^ Variants on Transcript and Protein Expression

				Effect of Mutation		
Patient	Age (Y)	Phenotype	Mutation	Sequence Alterations	mRNA Level	Protein Level
1	35	Central macular atrophy with far inferior peripheral atrophy	c.2027_2039del (p.Lys676Thrfs*17)	Frameshift; new cryptic acceptor site; significant alteration of ESE/ESS motifs ratio	Premature stop codon; exon skipping	Complete or partial loss of function
234	364245	Central macular atrophy with inferior arcade and nasal retinal atrophy	c.2405_2406delAG (p.Glu802Glyfs*32)	Frameshift	Premature stop codon	Complete or partial loss of function
5	51	Central macular atrophy with inferior retinal atrophy	c.2426_2427delAG (p.Glu809Glyfs*25)	Frameshift	Premature stop codon	Complete or partial loss of function
6	14	Central macular atrophy with inferior retinal atrophy	c.3040delG (p.Glu1014Argfs*75)	Frameshift	Premature stop codon	Complete or partial loss of function
7^*^89^*^10^*^11^*^12	496469727591		c.3092delA (p.Glu1031Glyfs*58)	Frameshift; new cryptic donor site; significant alteration of ESE/ESS motifs ratio	Premature stop codon; exon skipping; abnormal exon inclusion	Complete or partial loss of function; dominant gain-of-function
13	53	Central macular atrophy with nasal retinal atrophy	c.3092_3093delAG (p.Glu1031Glyfs*47)	Frameshift; new cryptic donor site	Premature stop codon; exon skipping; abnormal exon inclusion	Complete or partial loss of function; dominant gain-of-function
14	71	Central macular atrophy with inferior arcade atrophy	c.3178_3179delGA (p.Glu1060Argfs*18)	Frameshift; new cryptic acceptor site	Premature stop codon; exon skipping;	Complete or partial loss of function

* Patients 7, 9, 10, and 11 belong to the same pedigree.

**Figure 3.**
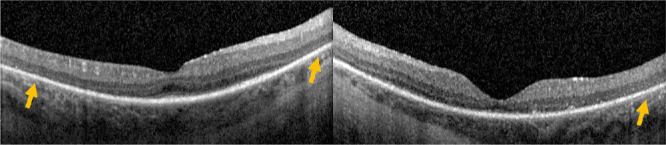
(**a**, **b**) OCT of the macula showing foveal and parafoveal outer retinal loss (*arrows*) in the right (**a**) and left (**b**) eyes.

**Figure 4.**
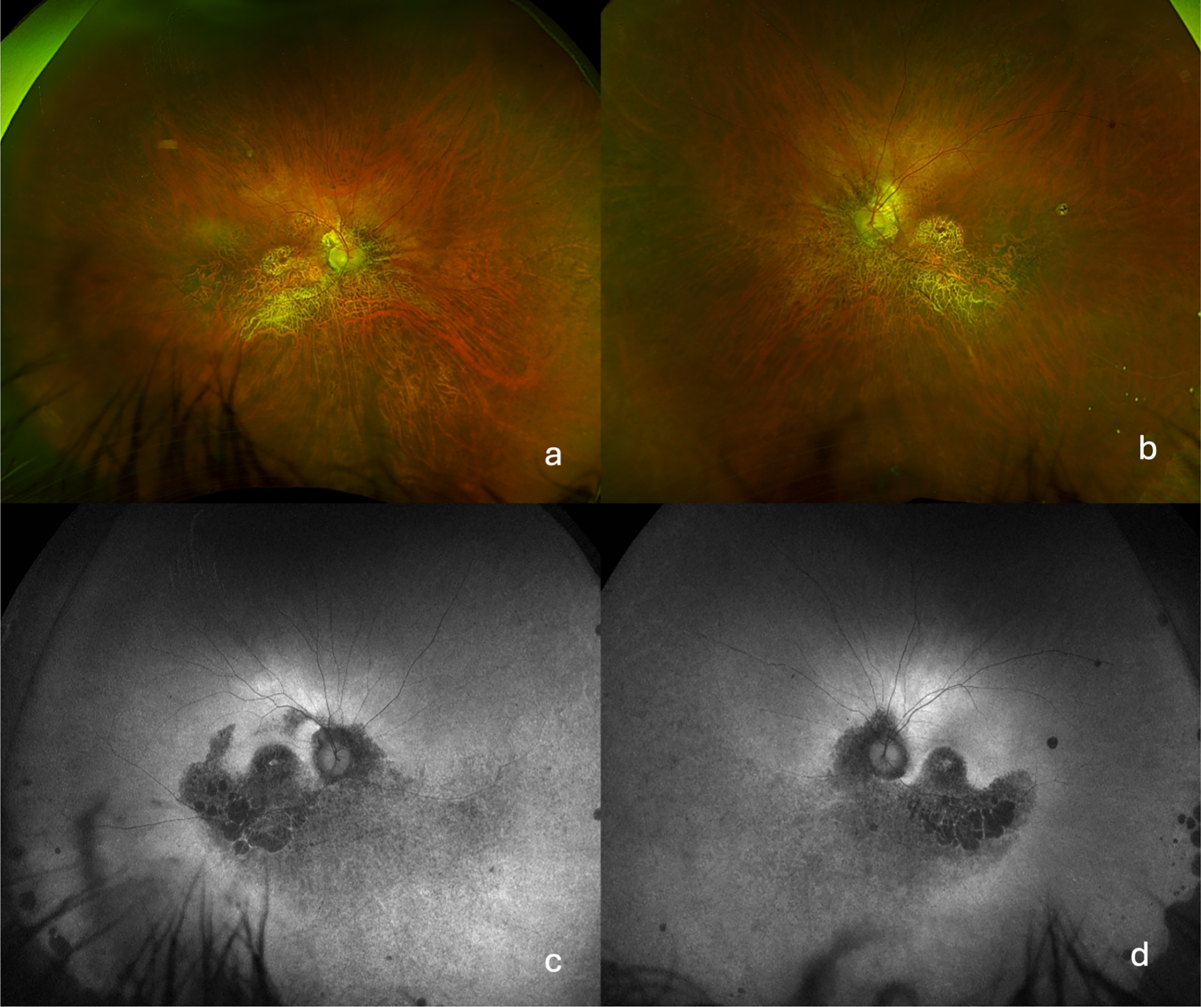
(**a**–**d**) Ultra-widefield pseudocolor fundus images demonstrating atrophy along the inferior arcades in the right (**a**) and left (**b**) eyes and corresponding autofluorescence images in the right (**c**) and left (**d**) eyes.

**Figure 5.**
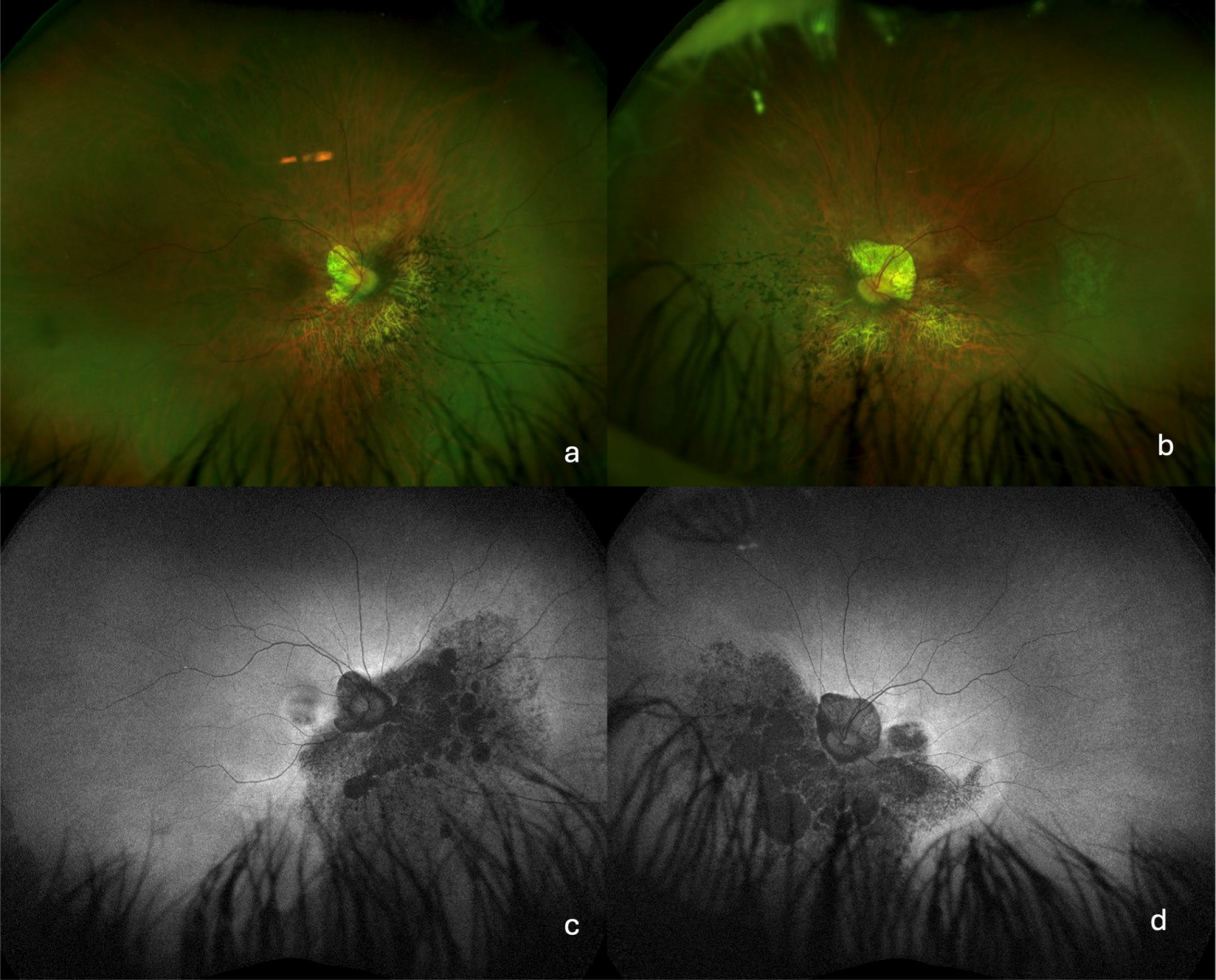
(**a**–**d**) Ultra-widefield pseudocolor fundus images demonstrating atrophy extending more nasally rather than along the inferior arcade in the right (**a**) and left (**b**) eyes and corresponding autofluorescence images in the right (**c**) and left (d) eyes.

**Figure 6.**
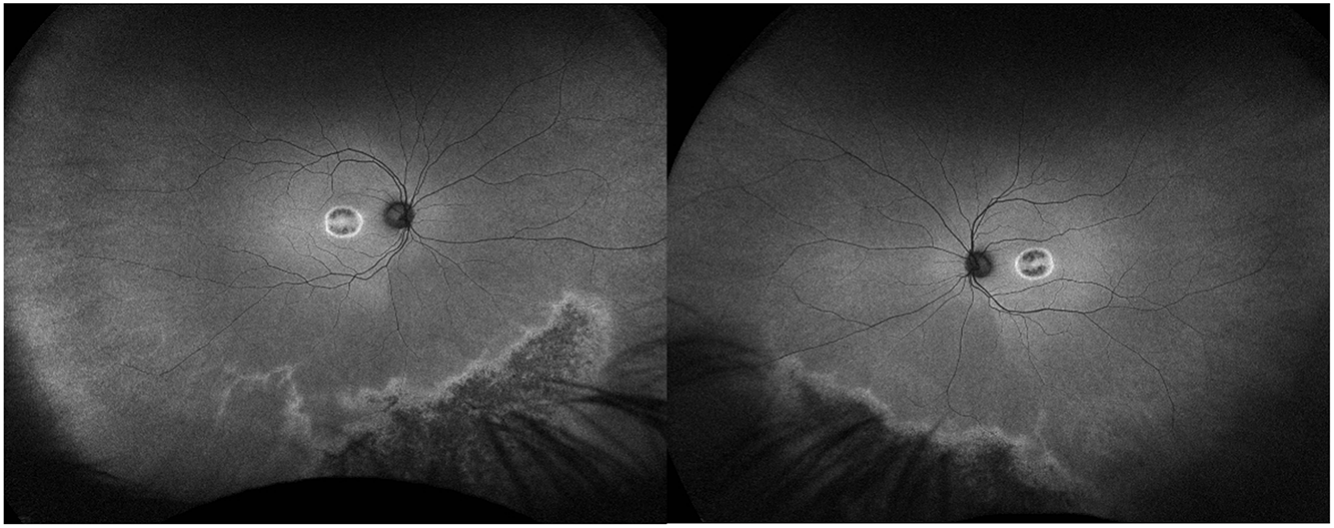
(**a**, **b**) Ultra-widefield autofluorescence images demonstrating far inferior peripheral retinal atrophy in the right (**a**) and left (**b**) eyes.

**Figure 7.**
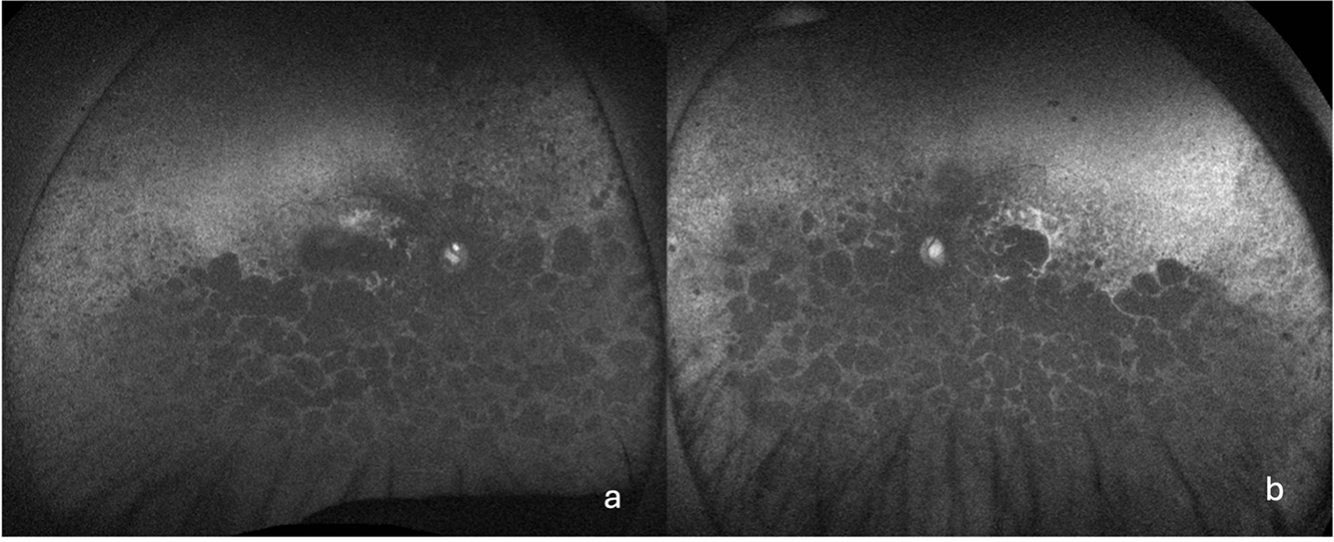
(**a**, **b**) Ultra-widefield autofluorescence images demonstrating generalized atrophy with much greater predilection for the macula and inferior retina in the right (**a**) and left (**b**) eyes.

**Figure 8.**
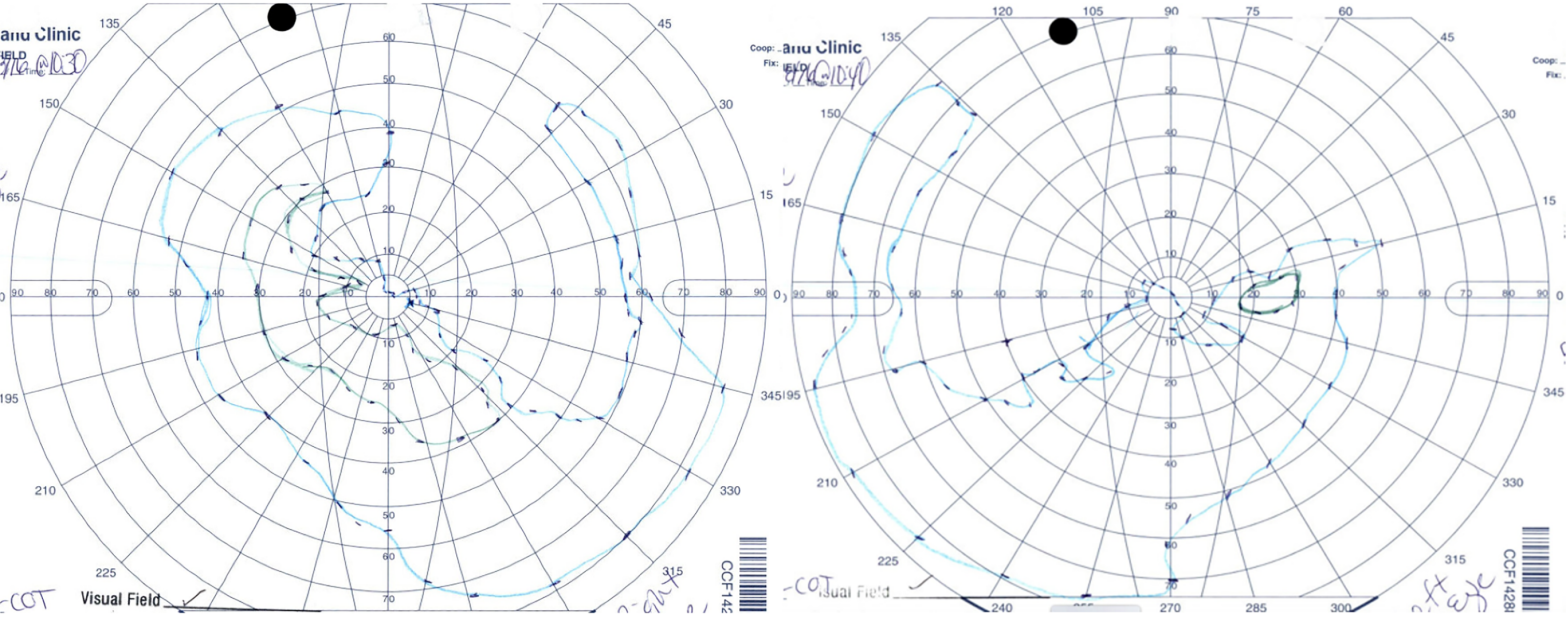
Goldmann visual fields demonstrating constriction of the superior visual fields.

Patients reported hemeralopia, photophobia, dyschromatopsia, and impaired central vision attributable to cone dysfunction. All patients had clinical features of a central cone degeneration with distinctly reduced visual acuity and central macular hypo-autofluorescence with or without a high-density hyper-autofluorescent perimacular ring on fundus autofluorescence (FAF). OCT demonstrated disruption of the parafoveal ellipsoid zone and central outer nuclear layers of different degrees consistent with a central cone degeneration (Fig. 3). Thirteen of the 14 patients had bilateral, symmetrical outer retinal atrophy located along the inferior vascular arcades (Fig. 4). These lesions extended more nasally in one patient, forming a crescent-shaped distribution (Fig. 5). In addition, seven of the 14 patients displayed marked peripheral photoreceptor degeneration as shown by the hypo-autofluorescent patches in the far-peripheral inferior retina (Figs. 6, 7). Visual field testing in two patients revealed superior visual field defects closely correlated with the areas of inferior retinal atrophy (Fig. 8).

Age did not appear to correlate with the severity of degeneration seen in the ophthalmoscopic phenotype as illustrated by four patients with the same mutation (*RPGR* c.3092delA, p.[Glu1031Glyfs*58]) and from the same pedigree who exhibited intermediate retinal findings. Interestingly, the youngest patient from this pedigree, a 49-year-old male, exhibited the most severe phenotype with dense inferior retinal degeneration extending from the inferior retinal arcades to the far periphery with no photoreceptor preservation in the intervening areas (Fig. 7). Nine patients had longitudinal data. None of these patients exhibited transition to a generalized rod–cone dystrophy phenotype. In addition, we report a patient who retained the sector rod–cone dystrophy phenotype up to the 10th decade of life. The longest follow-up period, spanning 12 years, involved a patient with the *RPGR* c.2405_2406delAG mutation. FAF images indicate progression in retinal degeneration; however, no progression to generalized rod–cone dystrophy was observed within the available follow-up.

Electroretinography was available for three patients. In the first patient, full-field ERG showed markedly reduced scotopic and maximal responses, undetectable photopic responses, and undetectable multifocal ERG responses across all rings. In the second patient, scotopic responses were absent, maximal responses were reduced and delayed, photopic responses were non-recordable, and 30-Hz flicker responses were small and delayed. In the third patient, ERG in 2006 showed grossly reduced and delayed scotopic responses, reduced maximal responses, reduced and significantly delayed photopic and 30-Hz flicker responses, and multifocal ERG responses that were undetectable in the superior field and reduced and delayed in the inferior field. Repeat testing in 2013 showed reduced and delayed scotopic, maximal, and photopic responses, greater in the left than right eye but essentially unchanged from 2006, with multifocal ERG again showing greater involvement of the superior field than the inferior field. These findings did not indicate significant electrophysiological progression over the 7-year interval.

### In Silico Analysis

**Figure 9.**
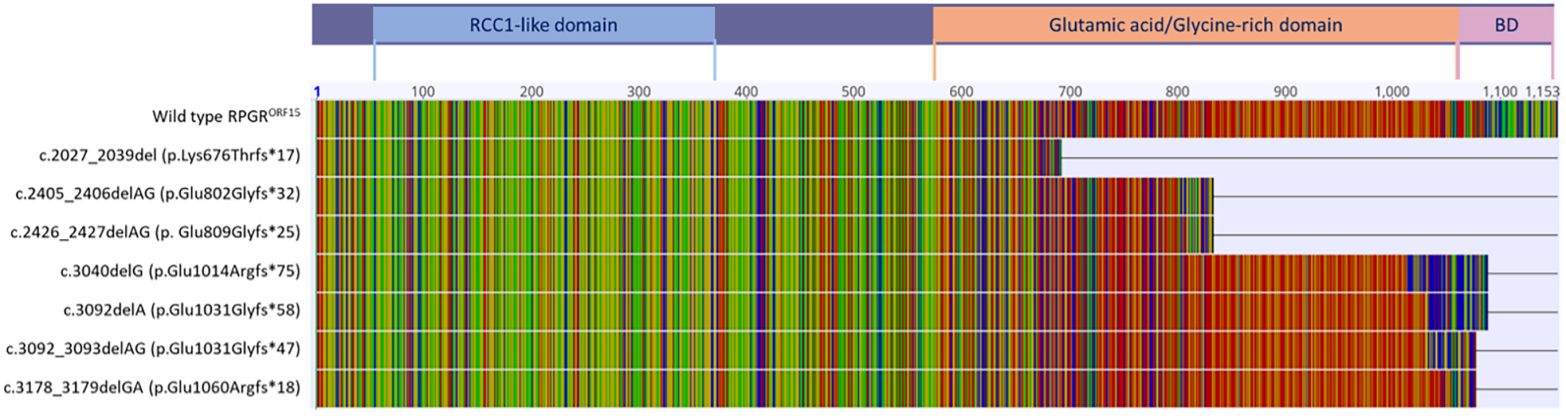
Amino acid composition of truncated RPGR^ORF15^ variants resulting from each mutation. The diagram above the alignment illustrates the domain structure of the full-length RPGR^ORF15^ protein. The alignment is color coded to show amino acid properties. *Yellow bars* indicate non-polar (hydrophobic) residues; *green bars*, polar, uncharged residues; *red bars*, polar, acidic (negative charge) residues; *blue bars*, polar, basic (positive charge) residues.

The mutations analyzed in this study involve deletions of one or more nucleotides, which cause a frameshift in the coding sequence during translation. This alteration disrupts the original reading frame, resulting in an entirely different downstream amino acid sequence and the introduction of premature stop codons. These variants escape NMD and remain stable for translation, as confirmed with NMDEsc predictor. As a result, truncated proteins are produced, which are often non-functional. Figure 9 shows a color-coded map representing the altered amino acid composition for each mutant variant. Specifically, the frameshift mutations c.3040delG, c.3092delA, c.3092_3093delAG, and c.3178_3179delGA (four bottom rows) resulted in the replacement of negatively charged residues (highlighted in red) with positively charged ones (in blue), affecting between 16 and 75 amino acids depending on the mutation.

To further understand the molecular impact of these variants, a series of bioinformatic analyses were conducted. These analyses provided insights into both transcript-level changes and potential disruptions to protein structure and function. A summary of the key sequence alterations, along with their predicted effects on transcript expression and protein integrity, is presented in the Table.

Two of the mutations, c.2027_2039del and c.3178_3179delGA, appear to activate a cryptic splice acceptor site located upstream of the deletion. This newly activated acceptor site may pair with the canonical donor site at the exon 15–intron boundary (around position 1906), leading to the exclusion of 100 to 300 nucleotides within the ORF15 region. Conversely, mutations c.3092delA and c.3092_3093delAG seem to activate a cryptic donor site upstream of the mutation site. This aberrant splicing event could have several consequences: It might lead to the inclusion of incorrect or non-coding exonic sequences into the mature transcript; disrupt mRNA splicing and cause exon skipping; or produce an unstable or misfolded protein. Such changes may either result in loss of normal protein function or present a gain-of-function effect. In particular, the activation of a cryptic donor site within ORF15 may result in the excision of the intervening sequence, from the cryptic site to exon 16, and generate a hybrid transcript composed of the 5′ portion of ORF15 followed by exons 16 through 19. This mosaic transcript would likely encode an aberrant protein with a distorted structure and altered function, potentially impairing the normal biological role of RPGR^ORF15^.

## Discussion

This study defines a novel, intermediate clinical phenotype of *RPGR*-associated retinal dystrophy consisting of cone dystrophy features, combined with sectoral RP-like features. Although uncommon, this phenotype is consistent with the lower prevalence of cone–rod presentations in RPGR-associated disease, which account for approximately 15% to 32% of reported cohorts.^2^^,^^17^ The observed phenotype is notable for a cone-dominant clinical course, evidenced by early central vision decline and preserved night vision in early stages. These patients have *RPGR^ORF15^* truncating mutations and are also likely to benefit from gene therapy involving the full-length RPGR^ORF15^ protein. As gene therapies for *RPGR*-related disease are entering clinical trials, understanding nuanced phenotypes will help tailor patient selection and inform treatment timing, although this intermediate phenotype is unlikely to substantially affect macular-based outcome measures.

This phenotype likely reflects an intermediate position within the known RPGR^ORF15^ genotype–phenotype gradient. Proximal variants are typically associated with rod–cone dystrophy, whereas distal variants affecting RPGR^ORF15^ glutamylation are linked to cone-dominated disease.^11^ The sectoral changes observed here may therefore represent partial distal protein dysfunction in near full-length RPGR^ORF15^. Additionally, this novel phenotype underscores the diagnostic importance of targeted Sanger sequencing of the *RPGR ORF15* region in patients presenting with such fundal features. Due to the low-complexity, purine-rich nature of ORF15, next-generation sequencing (NGS) technologies often exhibit reduced fidelity in this region, increasing the risk of false-negative results and potentially leading to missed molecular diagnoses.

Despite the limited longitudinal imaging data, cross-sectional assessment of such a cohort provides insights into the natural history of the disease in terms of both OCT and FAF findings. To control for bias caused by age-related progression or different mutations, phenotypic comparisons were also performed within a single pedigree. Notably, in our cohort, age did not appear to significantly influence the observed structural phenotype, and the persistence of this pattern in predominantly middle-aged and older patients supports that it may represent a stable clinical presentation rather than merely an early stage of progression toward generalized rod–cone dystrophy. The presence of this phenotype in predominantly middle-aged and older patients supports that it represents a persistent clinical pattern rather than merely an early stage of progression toward generalized rod–cone dystrophy. Nevertheless, we acknowledge that age-related progression or slower evolution of a cone-dominant phenotype could contribute to sectoral degeneration in some older patients.

Truncating variants at the distal end of *RPGR^ORF15^* are primarily associated with cone-dominated retinal dystrophies and have been shown to disrupt the interaction between RPGR^ORF15^ and the tubulin glutamylase TTLL5, leading to reduced glutamylation of the RPGR^ORF15^ protein.^11^ Truncated variants of the RPGR^ORF15^ protein are associated with a severe retinal phenotype, frequently mediated by a gain-of-function mechanism.^18^ The accompanying sectoral rod–cone dystrophy observed in these cases may thus reflect a gain-of-function effect that disrupts the function of both rod and cone photoreceptors. Sector retinitis pigmentosa is most frequently associated with autosomal dominant mutations, particularly in the *RHO* gene, encoding the photoreceptor protein rhodopsin. These mutations—most often located in the extracellular (intradiscal) domain—include p.Thr17Met (T17M), p.Gly106Arg, and p.Pro23His, and are associated with toxic protein misfolding, intracellular aggregation, and dominant-negative effects that disrupt photoreceptor integrity, leading to autosomal dominant rod–cone dystrophy.^19^^–^^21^ Beyond *RHO*, gain-of-function mechanisms have also been implicated in *RPGR*. The c.2091_2092insA (p.A697fs) RPGR mutation, for example, has been linked to pathologic myopia in both male patients and heterozygous females, suggesting a role in retinal remodeling.^2^^,^^22^^,^^23^ It is therefore plausible that mis-spliced *RPGR* transcripts could exert gain-of-function effects contributing to a rod dystrophy phenotype, alongside cone dysfunction due to C-terminal truncation. Taken together, these molecular consequences offer a mechanistic framework for understanding the severe cone-dominant phenotype associated with truncating mutations in the distal *RPGR^ORF15^* region, highlighting the complexity of genotype–phenotype correlations in X-linked retinal dystrophies. Patients enrolled in *RPGR* gene therapy trials in which a truncated RPGR is expressed with aberrant splice products should therefore be monitored more closely over the long term.^24^ These patients may also be at risk of a similar phenotype of accelerated rod degeneration due to dominant negative effects of truncated RPGR proteins.^25^

The phenotype characterized in this study closely resembles a loss-of-function *TTLL5*-related cone dystrophy with sectoral rod–cone involvement, suggesting possible shared pathophysiological mechanisms affecting the photoreceptor ciliary transport system.^26^ The overlap with TTLL5-associated disease may reflect convergence on impaired RPGRORF15 glutamylation rather than identical genetic mechanisms. In TTLL5 deficiency, loss of the glutamylase may impair post-translational modification of otherwise full-length *RPGR^ORF15^*^,^ whereas distal *RPGR^ORF15^* variants may disrupt the C-terminal region required for *TTLL5* binding and subsequent glutamylation. Both mechanisms could therefore impair *RPGR^ORF15^*-dependent ciliary trafficking, providing a plausible explanation for the shared cone-dominant phenotype with sectoral rod involvement, although this remains hypothetical.

The preferential involvement of the inferior and temporal vascular arcades, as well as regions nasal to the optic disc, in *RPGR*-associated retinal dystrophy likely reflects an underlying anatomical and physiological vulnerability informed by regional variations in photoreceptor density, metabolic demand, and structural support. Topographic mapping of the human retina by Curcio et al.^27^ demonstrated that rod photoreceptors are most densely distributed in an elliptical ring approximately 3 to 5 mm from the fovea, with peak densities located superiorly and nasally, and with extensions into the inferior and temporal periphery. Concurrently, a horizontal “cone streak” traverses the temporal and nasal retina, with relatively higher cone densities nasally and inferiorly than in the temporal and superior retina at equivalent eccentricities.^27^^,^^28^ The convergence of these two structural features—high rod density in the parafoveal ring and elevated cone density in the horizontal streak—results in regions of overlapping photoreceptor specialization, notably within the inferior and temporal arcades and nasal peripapillary retina. These regions are presumed to be metabolically demanding due to the dual burden of high rod and cone turnover, both of which rely on efficient ciliary trafficking of phototransduction proteins. In the context of *RPGR^ORF15^*-related dysfunction, which impairs microtubule-based intra-flagellar transport and outer segment renewal, these overlapping high-density zones may represent areas of early structural vulnerability.^5^

The situation may be further compounded by regional differences in choroidal perfusion and structural support. OCT studies have shown that choroidal thickness and choriocapillaris vascular density vary topographically across the posterior pole, with relative thinning and perfusion deficits in the inferior and nasal quadrants.^29^ These vascular features may limit the ability of the outer retina to meet the oxygen and nutrient demands imposed by dense photoreceptor populations in these regions. Collectively, the anatomical overlap of high photoreceptor density, elevated regional metabolic demand, and relative microvascular insufficiency offers a compelling anatomical and physiological framework for understanding the sectoral atrophy patterns observed in this *RPGR*-associated phenotype. Recognizing region-specific vulnerability—guided by detailed photoreceptor topography—may have important implications for clinical trial design, particularly in the selection of imaging endpoints and the monitoring of disease progression.

A limitation of this study is the absence of systematic full-field ERG data for the entire cohort. This reflects the retrospective multicenter design and current practice in many specialized IRD centers, where electrophysiology is not routinely performed when multimodal imaging provides sufficient phenotypic characterization.
